# PHC Progression Model: a novel mixed-methods tool for measuring primary health care system capacity

**DOI:** 10.1136/bmjgh-2019-001822

**Published:** 2019-09-13

**Authors:** Hannah L Ratcliffe, Dan Schwarz, Lisa R Hirschhorn, Cintia Cejas, Abdoulaye Diallo, Ezequiel Garcia-Elorrio, Jocelyn Fifield, Diane Gashumba, Lucy Hartshorn, Nicholas Leydon, Mohamed Mohamed, Yoriko Nakamura, Youssoupha Ndiaye, Jacob Novignon, Anthony Ofosu, Sanam Roder-DeWan, Angelique Rwiyereka, Federica Secci, Jeremy H Veillard, Asaf Bitton

**Affiliations:** 1 Ariadne Labs, Brigham and Women's Hospital & Harvard T.H. Chan School of Public Health, Boston, Massachusetts, USA; 2 Department of Medical Social Sciences, Northwestern University Feinberg School of Medicine, Chicago, Illinois, USA; 3 Secretaria de Gobierno de Salud, Ministerio de Salud y Desarrollo Social, Buenos Aires, Argentina; 4 Directorate of Planning, Research and Statistics, Ministry of Health and Social Action, Dakar, Senegal; 5 Institute for Clinical Effectiveness and Health Policy, Buenos Aires, Argentina; 6 Ministry of Health, Kigali, Rwanda; 7 Bill & Melinda Gates Foundation, Seattle, Washington, USA; 8 Ministry of Health, Community Development, Gender, Elderly, and Children, Dodoma, United Republic of Tanzania; 9 Results for Development, Washington, District of Columbia, USA; 10 Kwame Nkrumah University of Science and Technology, Kumasi, Ghana; 11 Division of Policy, Planning, Monitoring and Evaluation, Ghana Health Service, Accra, Ghana; 12 Health Section, UNICEF Tanzania, Dar es Salaam, United Republic of Tanzania; 13 Global Health Issues and Solutions, Kigali, Rwanda; 14 Health, Nutrition and Population Global Practice, World Bank Group, Washington, District of Columbia, USA

**Keywords:** Primary care, primary health care, measurement, capacity, global health, universal health coverage

## Abstract

High-performing primary health care (PHC) is essential for achieving universal health coverage. However, in many countries, PHC is weak and unable to deliver on its potential. Improvement is often limited by a lack of actionable data to inform policies and set priorities. To address this gap, the Primary Health Care Performance Initiative (PHCPI) was formed to strengthen measurement of PHC in low-income and middle-income countries in order to accelerate improvement. PHCPI’s Vital Signs Profile was designed to provide a comprehensive snapshot of the performance of a country’s PHC system, yet quantitative information about PHC systems’ capacity to deliver high-quality, effective care was limited by the scarcity of existing data sources and metrics. To systematically measure the capacity of PHC systems, PHCPI developed the PHC Progression Model, a rubric-based mixed-methods assessment tool. The PHC Progression Model is completed through a participatory process by in-country teams and subsequently reviewed by PHCPI to validate results and ensure consistency across countries. In 2018, PHCPI partnered with five countries to pilot the tool and found that it was feasible to implement with fidelity, produced valid results, and was highly acceptable and useful to stakeholders. Pilot results showed that both the participatory assessment process and resulting findings yielded novel and actionable insights into PHC strengths and weaknesses. Based on these positive early results, PHCPI will support expansion of the PHC Progression Model to additional countries to systematically and comprehensively measure PHC system capacity in order to identify and prioritise targeted improvement efforts.

**Summary box**

- Effective primary health care (PHC) is essential for achieving the promise of quality universal health coverage, but PHC performance is weak in many low-income and middle-income countries (LMICs).
- The ability to improve PHC systems is limited by a lack of relevant metrics and data for assessing critical areas of PHC performance and system capacity.
- The Primary Health Care Performance Initiative (PHCPI) undertook a structured, participatory process to design a new mixed-methods assessment tool to measure PHC capacity in LMICs more systematically and comprehensively.
- PHCPI partnered with governments in five LMICs to pilot the assessment tool in 2018 and found that the assessment yielded novel and actionable information on PHC strengths and weaknesses and was feasible, acceptable, and effective in generating local ownership of the results.

## Introduction

The 2018 Astana Declaration reaffirmed the global community’s commitment to strengthening primary health care (PHC) and highlighted the central role that PHC must play in the achievement of universal health coverage.^1^ However, too often PHC is weak, underprioritised, and unable to deliver on this potential, particularly in low-income and middle-income countries (LMICs).^2–4^

**Figure 1:**
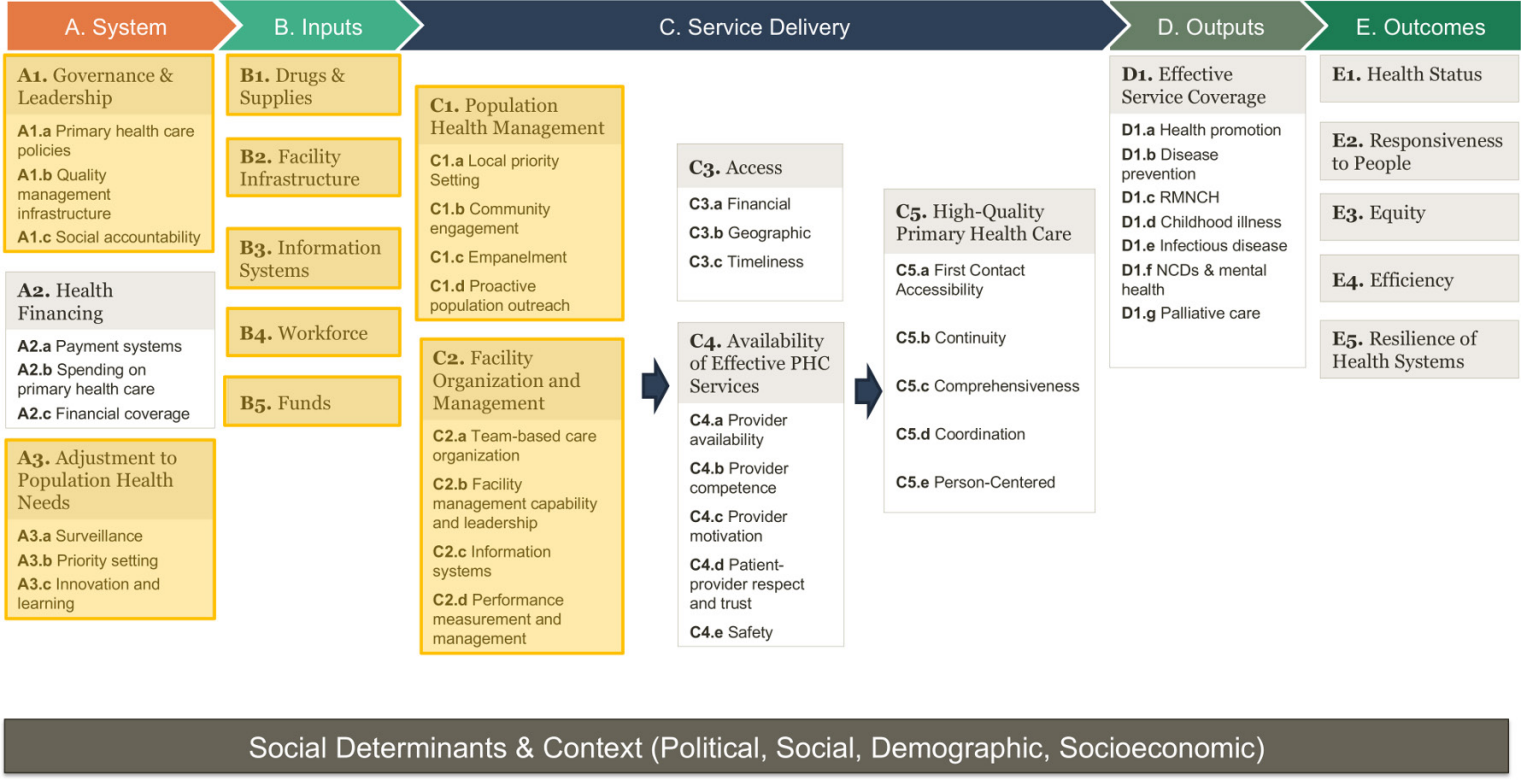
The Primary Health Care Performance Initiative conceptual framework. Focal areas for the PHC Progression Model are highlighted in yellow; other areas of the framework are also included in the Vital Signs Profile but assessed using different a methodology, reflecting available quantitative data. PHC, primary healthcare; NCDs, non-communicable diseases; RMNCH, reproductive, maternal, newborn, and child health.

In many LMICs, the ability to improve PHC is limited by a lack of relevant data to accurately measure and diagnose performance to inform policies and set priorities. The Primary Health Care Performance Initiative (PHCPI) was formed to accelerate improvements in PHC through better measurement and knowledge sharing.^5^ To guide its work, PHCPI developed a conceptual framework outlining the core systems, inputs, and service delivery elements necessary to produce strong PHC outputs and outcomes (figure 1)^6^ and conducted an extensive scoping process to identify the best globally available metrics to assess each component captured in the conceptual framework. Through these efforts, PHCPI was able to identify adequate measures for many components of the framework, including topics such as PHC spending, access, quality, service coverage, and health outcomes.^7^

However, PHCPI’s scoping process failed to identify robust, globally available indicators of the underlying capacities of PHC that impact overall performance and outcomes (figure 1).^7^ As described further in box 1, PHC capacities include topics such as:

Box 1 **Key questions assessed by the primary health care (PHC) Progression Model**

**Governance and leadership**

- Do countries have evidence-based PHC policies and strategies in place?
- Are there effective governance structures to implement and enforce these PHC policies?
- Is there a robust quality management infrastructure for PHC, including quality policies and strategies, legislation and regulation, quality standards, and use of continuous quality improvement programmes and methods?
- Does the country have a system that formalises and ensures strong social accountability mechanisms, including the systematic engagement of private sector, civil society, non-governmental organisations, and non-health actors in the integrated planning and governance of PHC and public disclosure of performance?

**Adjustment to population health needs**

- Do countries have comprehensive and reliable surveillance systems in place to detect and respond to changing disease burden and emerging outbreaks?
- Are national health priorities set based on disease burden, health outcomes, and user needs?
- Does the PHC sector have a learning system that prioritises continual reflection and improvement?

**Inputs**

- Are key inputs—including drugs and supplies, facility infrastructure, information systems, health workforce, and funds at the facility level—available?
- Are they equitably distributed?
- Are they of sufficiently high quality to meet population health needs?

**Population health management**

- Are local priorities evidence based and determined in collaboration with local communities and stakeholders?
- Do communities have input to and impact on the way that PHC is financed, governed, and implemented?
- Is a system of empanelment, or rostering, in place to ensure that the entire population is known to the health system and that specific service providers have responsibility for specific panels of patients?
- Does proactive population outreach occur to deliver essential health services to those in need?

**Facility organisation and management**

- Are primary care services organised and delivered by effective provider teams, capable of ensuring comprehensive and coordinated care?
- Are facilities effectively led by managers with the ability to organise operations, motivate staff, and deploy resources?
- Do facilities set performance targets, have staff capacity to capture and use data at the point of care to monitor and improve performance, and implement quality improvement activities?
- Is supportive supervision routinely conducted?

- How well the PHC system is governed and led.
- Whether PHC systems have the ability to detect and adjust to changing population health needs.
- Whether key inputs are available, equitably distributed, and of sufficiently high quality to meet population health needs.
- Whether PHC systems know and engage with the populations they serve.
- Whether healthcare providers work as teams in well-managed facilities, using data to drive improvement efforts.

Many of these are complex, interrelated topics whose performance varies over a continuum that is difficult to quantify and requires more nuanced exploration than a single quantitative measure may allow.

Through engagement and partnership with representatives from more than 30 LMICs over several years, PHCPI recognised that the collective inability to fully measure PHC capacity severely curtailed countries’ ability to develop a holistic understanding of their system’s strengths and weaknesses and hence their ability to identify and implement needed improvements. To address this measurement gap and ensure that essential information on PHC capacity could be comprehensively assessed, PHCPI developed a novel tool—the PHC Progression Model—to systematically assess the complex, foundational capacities of PHC. The results of the PHC Progression Model assessment are incorporated into the PHC Vital Signs Profile, a PHCPI measurement tool that summarises a country’s performance across the conceptual framework and is designed to support countries in identifying priority areas for improvement in PHC, track progress over time, and promote accountability for results by making essential performance information transparent and publicly available.^8^

In 2018, PHCPI partnered with five LMIC to pilot PHC Progression Model assessments. This paper describes the methods used to develop the PHC Progression Model as well as the process and lessons learnt from implementation.

## A new way of measuring PHC capacity

To identify a means of systematically assessing PHC capacity, PHCPI built on the recent increase in use of mixed-methods, rubric-based assessment tools in the health landscape. These tools are designed to capture performance across a range of different levels of system maturity, as defined by prespecified performance categories described in a series of rubrics. As a first step in conceptualising the PHC Progression Model, we used literature reviews, internet searches, expert recommendations, and snowball sampling to search for rubric-based tools in the healthcare domain. We identified a multitude of relevant tools, and six in particular that substantially influenced the conceptualisation and design of our work.^9–14^ Through a review of publicly available materials and interviews with the developers and/or implementers of these tools, we extracted relevant key learnings and the best practices around tool design and implementation (box 2). Additional information about these tools is available in online supplementary file 1.

10.1136/bmjgh-2019-001822.supp1 Supplementary data

Box 2 **Relevant lessons learnt on mixed-methods, rubric-based tool design and implementation from a review of existing tools**

- Rubric and standards-based tools can be used to assess across multiple, related domains.
- Most assessment tools employ a four or five-level categorisation, which enables nuanced descriptions of performance levels and/or progressive levels of maturity that can more completely capture complex topics and progression over time than simple binary indicators.
- These tools can be completed based on user opinion, qualitative data, quantitative data, document review and/or combinations of any of the above. Assessment can be internally driven by the stakeholders who participate in the activity being assessed or conducted by an objective, external body. Decisions around which methodology(ies) to use should be driven by the objectives of the assessment—for example, for comparison across sites or internal quality improvement purposes.
- The time and resources required to complete assessments are closely linked to the methods used.
- The assessment tool can be a vehicle for bringing together diverse stakeholders whose work relates to the same topic or output, but who may not have the opportunity to regularly interact with and learn from one another.

Based on these lessons learnt, PHCPI decided that the new assessment tool should be implemented through a joint internal and external assessment process led by in-country teams who rigorously document findings to collectively determine scores that are subsequently reviewed by an external team to ensure that results are rooted in evidence and that performance standards are consistently applied across countries. Such a process offered the best means of achieving PHCPI’s two goals for the assessment: (1) to produce a national assessment of PHC capacity that is acceptable to and owned by policymakers in LMIC and used to drive improvement efforts and (2) to drive accountability and improvement through the public release of Vital Signs Profiles that are standardised across countries and enable peer-to-peer benchmarking and learning.

## Development of the tool

**Figure 2:**
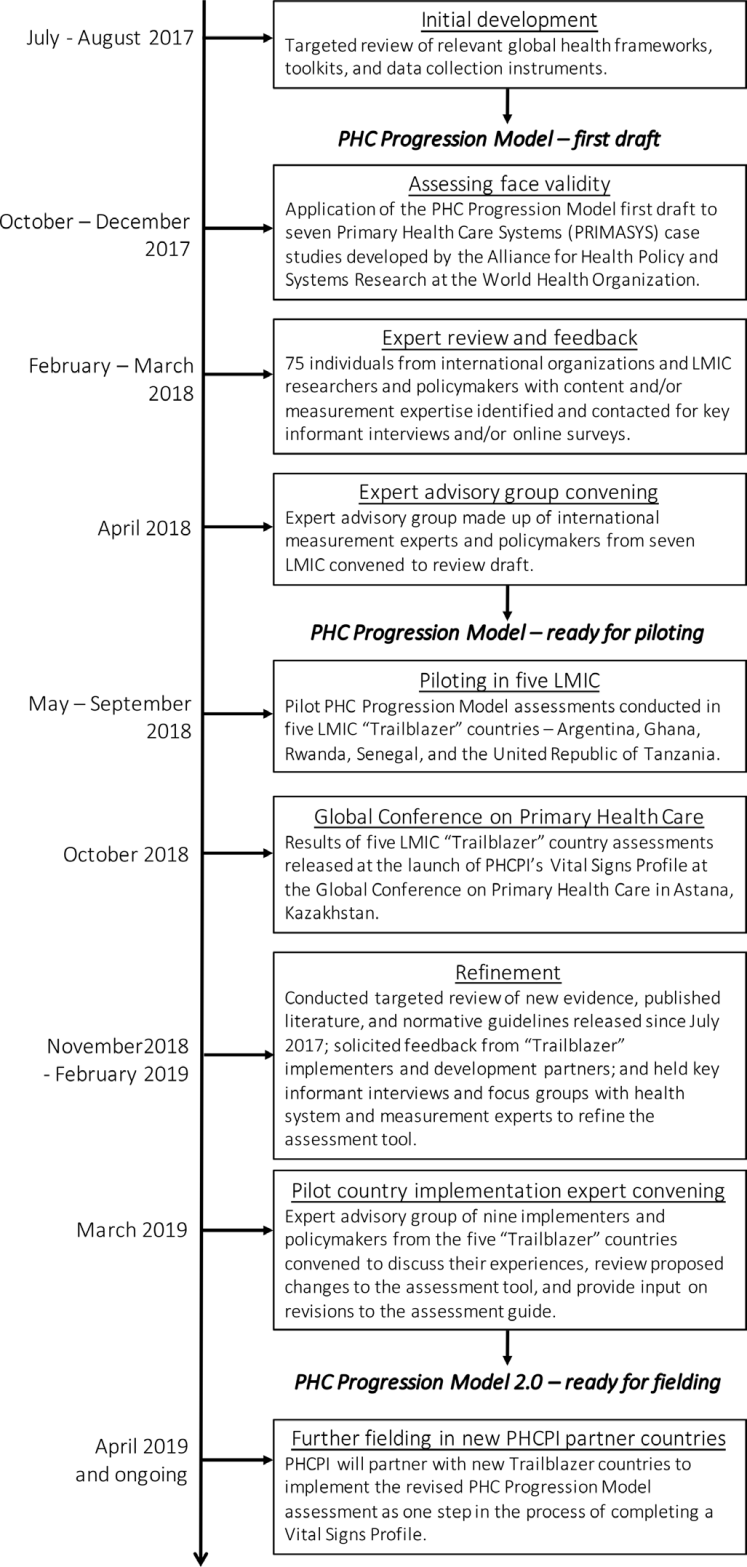
Timeline of the development of the PHC Progression Model. LMIC, low-income and middle-income country; PHC, primary healthcare; PHCPI, Primary Health Care Performance Initiative.

PHCPI undertook a structured, iterative, and participatory process to develop the PHC Progression Model (figure 2). The first step was a targeted review of relevant global health frameworks, toolkits, and data collection instruments^15–20^ to identify any indicators or normative standards related to the content to be assessed via the PHC Progression Model. Drawing where possible from these validated tools and supplementing with qualitative indicators to create a set of rubrics outlining four progressive performance categories for each topic assessed, we completed a first draft of the PHC Progression Model. To assess the face validity of the tool, we then partnered with the Alliance for Health Policy and Systems Research at the World Health Organization (WHO) to complete mock assessments using the seven Primary Health Care Systems case studies that were available at the time (Bangladesh, Ethiopia, Ghana, Kenya, Nigeria, Tanzania and Uganda).^21^ Based on the findings of this exercise, we made adjustments and generated a second draft of the tool.

Next, we identified 75 individuals with expertise in specific areas covered by the PHC Progression Model and conducted key informant interviews and online surveys. Each expert was asked to review the sections of the tool relevant to their area of expertise and reflect on the following dimensions of each measure: relevance, calibration, reliability, and comprehensiveness.

Finally, we convened an expert advisory group made up of international measurement experts as well as policy-makers from seven LMICs to review the content of the PHC Progression Model and assess whether each measure included in the tool captured the right information, was clear and well calibrated, and was feasible to assess in the represented countries. Based on all expert input received, we updated the PHC Progression Model to a version ready for pilot testing.

## The PHC Progression Model

**Figure 3:**
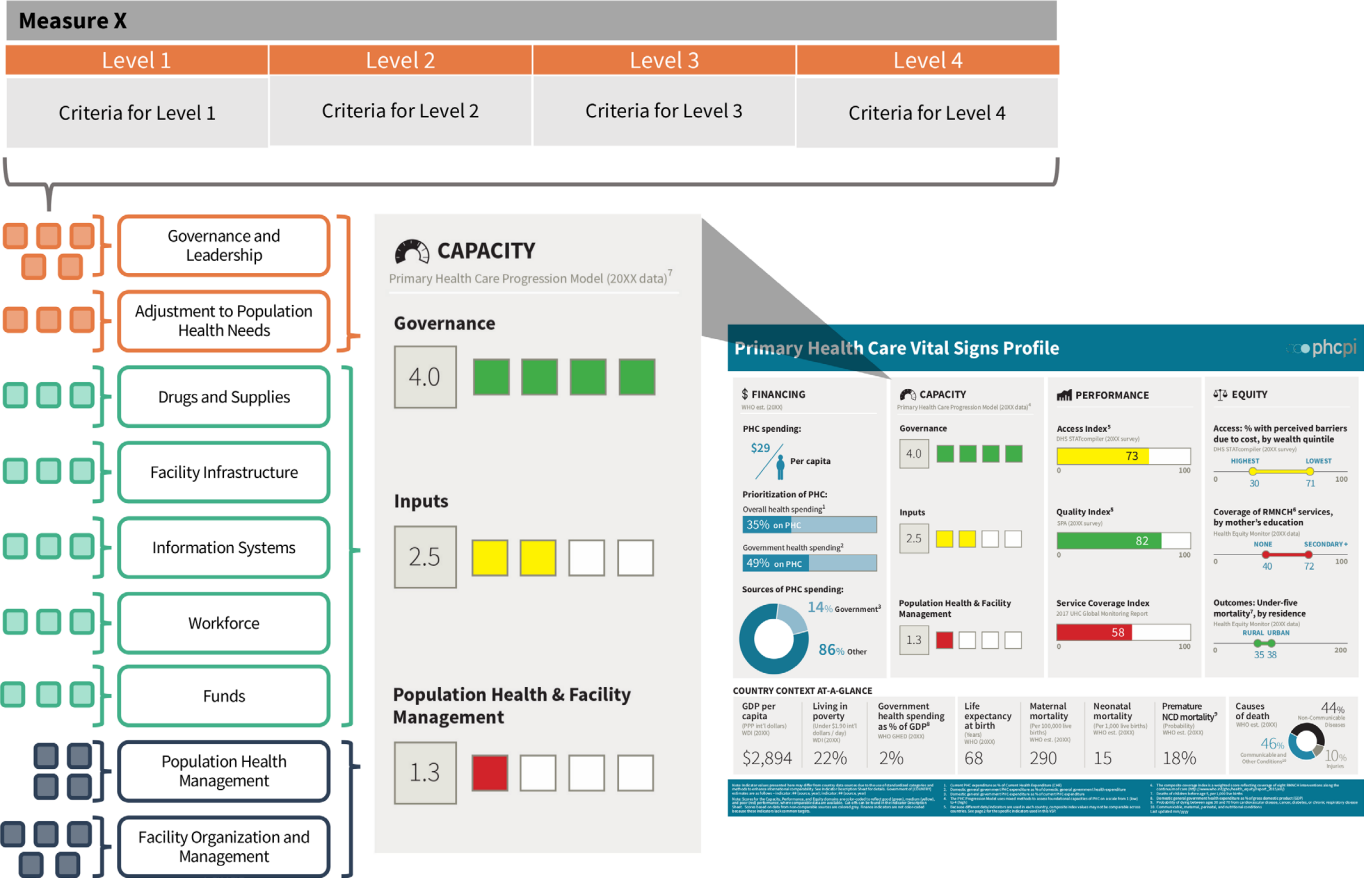
Structure of the PHC Progression Model and its relationship to the Vital Signs Profile (VSP). Each of the 32 measures of the PHC Progression Model contains a rubric outlining four performance categories (Levels 1–4). Measures are grouped thematically, according to the PHCPI conceptual framework. Raw measure scores are averaged by theme into nine subscores, which are in turn averaged to calculate the three scores that appear in the Capacity pillar of the VSP. Subscores and VSP scores are rounded to the tenths place. PHC, primary healthcare; PHCPI, Primary Health Care Performance Initiative.

The resulting PHC Progression Model is a mixed-methods assessment tool for measuring the foundational capacities of PHC.^22^ The model consists of 32 measures covering the content areas described in box 1. As shown in figure 3, each measure includes a rubric that is used to assign a country to one of four performance categories ranging from level 1 (low) to level 4 (high). The criteria for levels 1–4 vary according to the content of each measure being assessed; individual criteria can be found in the assessment tool.^22^ Data and evidence for completing the assessment can be drawn from a variety of sources available within countries, including policy documents; routine reports and assessments; data elements from global surveys and/or locally owned and generated data; and key informant interviews with a variety of public and private, governmental and non-governmental organisations including at the subnational levels, as appropriate depending on the measure to ensure inclusion of diverse perspectives.

Scoring of each measure of the PHC Progression Model employs a threshold approach, in which a performance level can only be achieved if all criteria described in the measure meet the performance described in the corresponding rubric. The 32 measure scores are summarised into nine subscores, corresponding to each subdomain of the PHCPI conceptual framework being assessed, by taking a simple unweighted average of all the constituent measures. The nine subscores are then summarised into three overall scores—Governance, Inputs, and Population Health and Facility Management—for display in the Capacity pillar of the Vital Signs Profile, again by taking simple unweighted average of the constituent subscores (figure 3).

## Piloting of the PHC Progression Model

PHCPI partnered with national Ministries of Health in five LMICs—Argentina, Ghana, Rwanda, Senegal, and the United Republic of Tanzania—to pilot a PHC Progression Model assessment in 2018. Countries were selected based on a formal expression of interest by the minister on behalf of their government, strong pre-existing relationship between one of the PHPCI partners and the ministry, and availability of resources to support the assessment.

Each country formed a core team responsible for implementing the assessment in accordance with the methods and standards outlined in a standardised assessment guide. As described in table 1, the size and composition of the core team varied by country depending on factors such as the time and availability of technical staff and preferences for inclusivity across multiple organisations or divisions within the Ministry of Health. Most often, the core team contained technical staff from Ministries of Health with expertise in and oversight of PHC in their country, as well as technical consultants who were often responsible for data collection.

**Table 1:** PHC Progression Model assessment strategies used across countries

	Argentina	Ghana	Rwanda	Senegal	Tanzania
External (PHCPI) team					
Lead organisation	World Bank Group	Results for Development	Bill & Melinda Gates Foundation	World Bank Group	World Bank Group
Context of relationships in country	Engaged via a US$300 million investment focused on supporting effective Universal Health Coverage.	Results for Development has a series of long-standing partnerships with governmental agencies in Ghana, primarily focused on health financing and health systems strengthening.	Technical partnership on projects related to telehealth and drone-delivered commodities.	Engagement with government through development of Global Financing Facility Investment Case and new World Bank Investment Project financing focused on improving maternal, child, and adolescent health.	Engaged via a US$200 million Program-for-Results focused on strengthening PHC system performance.
In-country team					
Ministerial engagement	Ministerial approval to initiate engagement. Active ministerial involvement and engagement throughout data collection and internal scoring exercise. Ministerial engagement to review results and approve release.	Ministerial approval to initiate engagement. Steering Committee made up of leaders from the MOH, Ghana Health Service, National Health Insurance Authority, and local representatives from the World Bank and WHO approved methods and findings. Ministerial engagement to review results and approve release.	Ministerial approval to initiate engagement. Minister of State engaged at assessment outset to approve process and give green light to conduct data collection. Active ministerial involvement and engagement throughout data collection and internal scoring exercise. Ministerial engagement to review results and approve release.	Ministerial approval to initiate engagement. Ministerial engagement to review results and approve release.	Ministerial approval to initiate engagement. Permanent Secretary engaged at assessment outset to approve process and give green light to conduct data collection. Ministerial engagement to review results and approve release.
Senior official engagement	Position: Under Secretary of Public Health Care Coverage, National Direction of Quality in Health Services and Health Regulations, and Office of the General Coordination Unit of the National MOH Role: Set direction of assessment, oversaw the composition of technical team, engaged as key informants, and provided political support throughout the process.	Position: Director of Policy, Planning, Monitoring and Evaluation within Ghana Health Service Role: Set direction of assessment, oversaw composition of technical team, engaged as key informant, and provided political support throughout the process.	Position: Director General of Planning, Health Financing and Information Systems and Director General of Rwanda Biomedical Center Role: Actively involved throughout data collection, engaged as key informants, provided political support, and participated in internal scoring exercise.	Position: Director of Planning, Research and Statistics Role: Set direction of assessment, oversaw composition of technical team, engaged as key informant, and provided political support throughout the process.	Position: Director of Department of Quality Assurance Role: Set direction of assessment, oversaw composition of technical team, engaged as key informant, and provided political support throughout the process.
Technical team	External, in-country consultant team made up of four researchers from the Institute of Clinical Effectiveness and Health Policy. TWG made up of the three MOH focal points, external consultants, and additional MOH staff and directors (four or more, depending on the topic under discussion), with support from in-country World Bank staff.	Two external, in-country doctoral-level quantitative and qualitative consultants Project coordinator. TWG led by Deputy Director of Policy, Planning, Monitoring and Evaluation of the Ghana Health Service and comprised representatives from: MOH University of Ghana Ghana Health Service Ghana Statistical Service National Health Insurance Authority UNICEF Ghana	Two external, in-country consultants, one PhD and one masters-level researcher. Working group led by Director of Monitoring and Evaluation and Rwanda Biomedical Center and the Health Information Systems and Report Lead Specialist at the MOH and comprised representatives from: MOH Rwanda Biomedical Center Rwanda Nursing Council District hospitals WHO Rwanda Office Management Sciences for Health and other development partners	Two external, in-country doctoral-level consultants Core working group led by General Director for Health Services, comprised representatives from: WHO Senegal Country Office World Bank Group Senegal National Statistics and Demography Agency Private Sector Alliance University and Research Institute	External, in-country doctoral-level consultant Working Group comprised: Representative from the MOH, Community Development, Gender, Elderly and Children National Professional Officer for Family and Reproductive Health of WHO Tanzania Country Office Senior Economist of the World Bank Group, Tanzania
Assessment process					
Preparation	PHCPI oriented consultants and provided overview of process.	TWG and Steering Committee formed. PHCPI oriented TWG and provided overview of process.	PHCPI oriented consultants and senior officials and provided overview of process.	PHCPI oriented consultants and senior officials and provided overview of process.	PHCPI oriented and provided overview of PHC Progression Model assessment process to consultants and working group.
Identification of data sources	Responsible person(s): Consultant team. MOH focal points reviewed and approved.	Responsible person(s): TWG.	Responsible person(s): Technical team. Working group reviewed and approved.	Responsible person(s): Core working group, senior MOH official and consultants.	Responsible person(s): Consultant and working group.
Data collection	Responsible person(s): Consultants 17 interviews completed. 22 documents reviewed. Regional/local perspective not available due to regulatory compliance and concurrent political events, so relevant parts of the assessment (PHFM) not completed at this time.	Responsible person(s): Qualitative consultant and TWG Approximately 70 interviews completed in total by the TWG and consultant. Approximately 40 documents reviewed. Regional/local perspective ensured by sampling a representative set of five regions and two districts (one urban and one rural) per region and conducting interviews with relevant regional and district staff. Quantitative data used came from reports and documents—no de novo analysis conducted.	Responsible person(s): Consultants 25 interviews completed. 105 documents reviewed and 10 main data websites consulted. TWG attended by 25 people including the MOH, the Director General of Rwanda Biomedical Center, the Director General in charge of planning, different Ministry’s department representatives, district hospitals directors, representatives of development partners, and local civil society organisations. Quantitative data used came from reports and documents—no de novo analysis conducted.	Responsible person(s): Consultants 22 interviews completed. 28 documents reviewed. Regional/local perspective ensured through attendance by TWG at scoring exercise.	Responsible person(s): Consultant 12 interviews completed by consultant. 25 documents reviewed by consultant. 8 datasets mined for quantitative data, including the STAR rating system, an accreditation system that collects data on an annual basis from all facilities. Regional/local perspective ensured by engagement with the President’s Office for Regional and Local Governance.
Data synthesis	Responsible person(s): Consultants	Responsible person(s): Qualitative consultant and programme coordinator. Support provided by Results for Development staff.	Responsible person(s): Consultants	Responsible person(s): Consultants	Responsible person(s): Consultant
Internal scoring	Consultants with input by MOH’s focal points.	TWG and consultants completed internal scoring.	Working Group and consultants convened to complete internal scoring.	Large TWG made up of 15 experts from across Senegal convened to offer evidence and perspectives through the internal scoring exercise.	Working Group and consultant convened to complete internal scoring. Workshop with broad representation from relevant MOH departments and partner organisations, including all who served as key informants, held to review internal scoring and provide additional input.

MOH, Ministry of Health; PHC, primary healthcare; PHCPI, Primary Health Care Performance Initiative; PHFM, Population Health and Facility Management; TWG, Technical Working Group.

The assessment team began by contextualising the assessment to the country context, a process which entailed agreeing to local definitions for: (1) the package of services considered to make up ‘PHC’, (2) the facilities considered to be ‘PHC facilities’ and (3) the human resources for health considered to be ‘PHC human resources’. Next, country teams undertook a detailed review of the types of data that would be required to score each measure, identified potential quantitative and qualitative sources, and made a plan for efficiently collecting needed data. When key informant interviews at the subnational level were deemed necessary, country teams also developed a sampling strategy to ensure that subnational data sources would yield a representative picture of what was truly occurring in the country.

Countries approached data collection in diverse ways, with some choosing to centralise the process within one or two individuals and others distributing responsibility to multiple members of the assessment team. Teams based decisions around considerations of feasibility, acceptability and effectiveness, including: how to structure their assessment process based on experience with similar assessment methods, for example, the Joint External Evaluation^12^; the time and resources key stakeholders could commit to the process; and an understanding of what steps would be necessary to generate local ownership of the results and be most likely to encourage the use of results to drive improvement efforts. Table 1 summarises the different assessment processes undertaken by each country team.

Data collection plans were approved by stakeholders such as high-level representatives from Ministries of Health and core implementing partners whose acceptance of the results would be critical for ensuring resulting data would be used to drive improvement efforts. Teams then completed data collection and used templates provided by the PHCPI team to compile and synthesise relevant information from across all data sources for each measure.

These data syntheses were then used as the evidence base for an internal scoring exercise, which consisted of convening a stakeholder group to review all of the assembled evidence for each measure and use the rubric to assign the country’s performance to one of the four performance categories. In the event that assessment teams had been unable to identify sufficient data to score a measure, a score of level 1 was assigned. (In Argentina, the federal administrative nature of the health system and current events made assessment of Population Health and Facility Management unfeasible, and results were instead displayed as ‘N/A’.) Participants in the scoring exercise were selected by the assessment team based on how best to generate buy-in for and acceptance of the results.

Next, the results of the internal scoring exercise, along with all supporting evidence, were shared with the PHCPI country engagement lead and a team from PHCPI partner organisation Ariadne Labs for external validation. The goals of the external validation were to ensure that the available evidence justified the scores given by the country team and that measurement standards were being consistently applied across countries. Often, the external assessment process resulted in the identification of measures where more detailed evidence was needed to justify the internal scores; in these cases, the external and country assessment teams would engage in ongoing dialogue and review of additional evidence until agreement was reached on the appropriate score. Final results were then integrated into the Vital Signs Profile, which was presented to the Minister of Health or equivalent for approval to be released. The typical length of time to complete the entire PHC Progression Model assessment process was approximately 3 months.

## Findings and lessons learnt

The PHC Progression Model is a novel tool for systematically assessing PHC capacity at a national level and provides a basis for countries to track their progress in creating better conditions for stronger performance over time. Additionally, due to its standardised methodology and structure, the PHC Progression Model and the Vital Signs Profile overall enables countries to engage in cross-country learning and peer-to-peer benchmarking if they choose, though neither tool is intended to be used for direct ranking or comparison purposes.

PHCPI conducted targeted outreach with implementers of the first five pilot assessments to collect their insights on the process and lessons learnt. Overall, the results of the five PHC Progression Model assessments (table 2) demonstrate that the process and methodology were feasible and acceptable. The measurement tool was able to be implemented with fidelity and to detect meaningful variation in PHC system capacity across and within countries. The tool also had internal validity, with internal and external scores being highly aligned—on average across all countries, 60% of measure scores were fully aligned across internal, external, and consensus scores while only 7% of scores differed by two or more performance levels between internal and final consensus scores. Only one measure had two or more countries differ by at least two performance levels from the internal to final consensus score. Importantly, not all differences between the internal and external scores shown in table 2 were due to PHCPI ‘correcting’ internal scores; in cases with misalignment, external scores were both higher and lower than internal scores, and it was typical that discussions about the difference between internal and external scores would surface implicit knowledge being applied by in-country teams and lead to the identification of additional data sources and evidence to justify the initial scores and resolve discrepancies.

**Table 2:** Comparison of internal, external and consensus scores across five pilot countries

VSP Domain	Subdomain	Measure	Argentina			Ghana			Rwanda			Senegal			Tanzania			Alignment of internal, external, and consensus scores (n, %)	Variation between internal score and consensus score of 2+ (n, %)
			I	E	C	I	E	C	I	E	C	I	E	C	I	E	C		
Governance			2.9	2.6	3.0	2.9	2.6	2.8	3.5	3.2	3.5	2.5	2.4	2.4	2.3	2.5	2.9		
	Governance and leadership		2.4	1.8	2.6	3.2	2.6	3.0	3.6	3.4	3.6	2.4	2.4	2.4	3.2	3.2	3.2		
		1: PHC policies (1/2)	2	2	2	4	3	3	4	4	4	2	3	3	3	4	3	2 (40)	0 (0)
		2: PHC policies (2/2)	1	1	2	3	2	3	3	3	3	2	2	2	3	3	3	3 (60)	0 (0)
		3: Quality management infrastructure	3	2	3	4	3	4	4	2	3	2	2	2	3	3	3	2 (40)	0 (0)
		4: Social accountability (1/2)	2	2	2	3	3	3	3	4	4	3	2	2	3	3	3	3 (60)	0 (0)
		5: Social accountability (2/2)	4	2	4	2	2	2	4	4	4	3	3	3	4	3	4	3 (60)	0 (0)
	Adjustment to population health needs		3.3	3.3	3.3	2.7	2.7	2.7	3.3	3.0	3.3	2.7	2.3	2.3	2.7	2.7	2.7		
		6: Surveillance	4	4	4	3	3	3	3	3	3	4	3	3	3	3	3	4 (80)	0 (0)
		7: Priority Setting	3	3	3	2	2	2	3	2	3	2	2	2	2	2	2	4 (80)	0 (0)
		8: Innovation and Learning	3	3	3	3	3	3	4	4	4	2	2	2	3	3	3	5 (100)	0 (0)
Inputs			2.0	1.7	2.7	2.3	2.3	2.4	3.1	2.6	2.6	2.4	2.1	2.1	1.8	1.3	2.2		
	Drugs and supplies		1.3	1.3	3.0	1.3	1.0	1.3	3.7	3.0	3.0	2.3	2.0	2.0	2.3	1.0	2.3		
		9: Stock-out of essential medicines and consumable commodities	1	1	4	1	1	2	4	3	3	1	1	1	3	1	2	1 (20)	1 (20)
		10: Basic equipment	2	2	2	1	1	2	4	3	3	3	3	3	2	1	3	2 (40)	0 (0)
		11: Diagnostic supplies	1	1	3	2	1	3	3	3	3	3	2	2	2	1	2	1 (20)	1 (20)
	Facility infrastructure		2.3	1.7	3.0	1.7	1.7	1.7	2.7	2.0	2.0	2.0	2.0	2.0	1.7	1.7	2.0		
		12: Facility Density	3	3	3	3	3	3	2	2	2	3	3	3	2	2	2	5 (100)	0 (0)
		13: Facility amenities	3	1	3	1	1	1	3	3	3	2	2	2	2	2	2	4 (80)	0 (0)
		14: Standard safety precautions and equipment	1	1	3	1	1	1	3	1	1	1	1	1	1	1	2	2 (40)	2 (40)
	Information systems		3.0	3.0	3.3	2.3	2.3	2.3	2.7	2.3	2.3	2.7	1.7	1.7	2.0	1.7	2.0		
		15: Civil registration and vital statistics	3	3	4	1	1	1	1	1	1	3	1	1	1	1	1	3 (60)	1 (20)
		16: Health management information systems	3	3	3	3	3	3	4	3	3	3	3	3	3	3	3	4 (80)	0 (0)
		17: Personal care records	3	3	3	3	3	3	3	3	3	2	1	1	2	1	2	3 (60)	0 (0)
	Workforce		2.3	1.3	2.0	3.0	3.0	3.3	2.7	1.7	1.7	2.5	1.7	1.7	1.3	1.3	1.3		
		18: Density and distribution	1	1	1	1	1	1	1	1	1	1	1	1	1	1	1	5 (100)	0 (0)
		19: Training	3	1	2	3	2	2	4	2	2	2	2	2	2	2	2	2 (40)	1 (20)
		20: Community health workers	2	1	2	3	4	4	3	2	2	3	2	2	1	1	1	1 (20)	0 (0)
	Funds		1.0	1.0	2.3	3.3	3.3	3.3	4.0	4.0	4.0	2.7	3.0	3.0	3.3	3.3	3.3		
		21: Facility budgets	1	1	3	3	3	3	4	4	4	2	3	3	3	3	3	3 (60)	1 (20)
		22: Financial management information system	1	1	3	3	3	3	4	4	4	3	3	3	3	3	3	4 (80)	1 (20)
		23: Salary payment	1	1	1	4	4	4	4	4	4	3	3	3	4	4	4	5 (100)	0 (0)
Population Health and Facility Management			--*	--	--	2.4	3.0	2.7	3.8	2.4	3.1	2.2	2.3	2.3	2.0	1.0	2.1		
	Population health management		--	--	--	2.0	3.0	2.5	3.5	2.5	3.3	2.3	2.3	2.3	2.3	1.5	2.0		
		24: Local priority setting	--	--	--	2	3	3	4	4	4	2	2	2	3	2	3	2 (50)	0 (0)
		25: Community engagement	--	--	--	3	4	3	4	3	3	2	2	2	2	2	2	2 (50)	0 (0)
		26: Empanelment	--	--	--	1	1	1	2	2	2	2	2	2	2	1	1	2 (50)	0 (0)
		27: Proactive population outreach	--	--	--	2	4	3	4	1	4	3	3	3	2	1	2	1 (25)	0 (0)
	Facility organisation and management		--	--	--	2.8	3.0	2.8	4.0	2.2	3.0	2.2	2.4	2.4	2.0	2.0	2.2		
		28: Team-based care organisation	--	--	--	3	3	3	4	1	2	2	2	2	1	1	1	3 (75)	1 (25)
		29: Facility management capability and leadership	--	--	--	2	2	2	4	1	2	2	2	2	2	2	2	3 (75)	1 (25)
		30: Information system use	--	--	--	3	3	3	4	1	3	2	2	2	2	2	2	3 (75)	0 (0)
		31: Performance measurement and management (1/2)	--	--	--	3	3	3	4	4	4	2	3	3	3	4	3	2 (50)	0 (0)
		32: Performance measurement and management (2/2): Supportive Supervision	--	--	--	3	4	3	4	4	4	3	3	3	2	1	3	2 (50)	0 (0)
Measures for which scores were aligned across internal, external, and consensus scores (n, %)			11 (46)			22 (69)			17 (53)			22 (69)			20 (63)				
Measures for which internal score varied by two or more performance levels from the consensus score (n, %)			5 (21)			0 (0)			4 (13)			1 (3)			0 (0)				

Colour coding: Red=score of 1.0–1.9; orange=score of 2.0–2.9; yellow=score 3.0–3.9; green=score of 4.0.

*Population health and facility management was not assessed in Argentina.

C, consensus score; E, external score;I, internal score; PHC, primary health care; VSP, Vital Signs Profile.

Overall, we found that completion of the PHC Progression Model assessment was a process which generated valuable new collaborations and insights for countries. The information needed to score individual measures and complete the assessment was often located within a multitude of documents and key informants. Bringing together all of these different data sources created a unique opportunity to collaboratively and holistically assess and understand an individual country’s PHC capacities in a way that is difficult if not impossible to do otherwise. This process of conducting the assessment and strategy of bringing together diverse stakeholders, each of whom had deep insight into a different piece of PHC, was often as valuable for understanding PHC capacity as the actual assessment results. Stakeholders reported that the process of implementing assessments made the identification of a system’s capacity strengths and weaknesses ‘glaringly obvious’ and that the assessment process resulted in learnings that ‘challenged pre-existing expectations’, even for stakeholders who had long been deeply embedded in the system. Since completion of assessments, all five country teams have initiated efforts to use the results of the PHC Progression Model and Vital Signs Profile to identify targeted improvement plans and inform efforts to expand data availability to more routinely measure areas assessed by the tool.

Our results also demonstrate that there are multiple implementation approaches that can be successfully employed to complete a PHC Progression Model assessment that is appropriate for the country context. Key core processes shared across countries that enabled success are summarised in box 3 and included contextualising the assessment within ongoing efforts by the government to improve PHC, obtaining both high-level ministerial buy-in and deep technical engagement, and customising the assessment strategy to meet local expectations and norms.

Box 3 **Implementation steps that enabled success across countries**

- Positioned within an ongoing strategic effort of the government to improve primary health care so that the assessment is not perceived as an ad hoc or standalone effort.
- Obtaining high-level buy-in and leadership from the ministerial level.
- Careful messaging of the assessment’s purpose as the first step in an improvement effort rather than as a punitive or audit tool.
- Fostering a participatory assessment process.
- Customising the assessment strategy—including working group composition, data collection strategies, and approach to the scoring exercise—to meet country expectations and norms.
- Deep engagement by in-country technical teams with ongoing, trusting relationships with government officials.
- Strong relationships between Primary Health Care Performance Initiative and the local assessment teams that established trust, mutual respect, and transparency.

The piloting of the PHC Progression Model identified limitations of the tool and assessment process. Most notably, the assessment was challenging to implement in a federalised country (Argentina) where the high degree of decentralisation meant that national-level data sources were unable to yield sufficient, timely information about on-the-ground realities across provinces. In federalised countries, subnational rather than national assessments of PHC capacity may be both more feasible to conduct and yield more informative results. Additionally, as noted above, completion of an assessment required investment of resources, including focused time from stakeholders and often necessitated hiring a consultant to support in-country efforts. We anticipate that repeat assessments in a country will be able to build on results and infrastructure established through the initial assessment, and therefore require less time and fewer resources. However, it will be critical for PHCPI to identify ways to streamline the assessment process and increase sustainability of the PHC Progression Model. Finally, the external validation process was designed to ensure that the collected data supported the scores proposed by country teams, however, external teams did not conduct quality reviews of data sources or any independent data collection efforts.

## Future directions

Results of each country’s assessment were released as part of their complete Vital Signs Profile at the Global Conference on PHC in Astana, Kazakhstan in 2018. Following the successful piloting of the PHC Progression Model, PHCPI undertook efforts—including expert consultations and a convening of early implementers—to refine the assessment tool to address any challenges identified during the pilot phase and ensure the measurement criteria in the tool reflect guidance and standards released since tool development began in 2017. In April 2019, PHCPI released an updated version of the assessment tool.^23^ Additionally, PHCPI updated the assessment guide to incorporate lessons learnt from the pilot experience; more information about the assessment guide is available on request.^22^ Moving forward, PHCPI has already initiated partnerships with ten additional countries—with many more planned—to expand the use of this assessment.

## Conclusion

Through a structured, participatory process, PHCPI developed and piloted a novel assessment tool to systematically measure PHC system capacity. Pilot assessments in five countries found the tool to be feasible to implement with fidelity, acceptable to stakeholders, and able to produce valid results. Both the assessment process and results were found to be highly valuable to country stakeholders and are now being used to inform improvement efforts. The PHC Progression Model is a promising new approach for generating comprehensive, standardised, and actionable data on PHC capacity to complement other key performance indicators and develop a holistic understanding of PHC strengths and weaknesses that can be used to drive improvement efforts.
