# Supplementary material for: PHC Progression Model: a novel mixed-methods tool for measuring primary health care system capacity
Source: BMJ Glob Health. 2019 Sep 13;4(5):e001822. doi: 10.1136/bmjgh-2019-001822 (PMC6748065; doi:10.1136/bmjgh-2019-001822)
Supplement: Supplementary data [file bmjgh-2019-001822supp001.pdf]

Assessment Tool	Links	Type of Assessment	Content Assessed	Tool Structure	Assessment Process	Resources required	Takeaways
**SCIROCCO**	https://www.scirocco-project.eu/maturitymodel/	Self	Regional readiness for implementing integrated care	Online.<br><br>12 domains, each assessed on a scale of 0-6.<br><br>Results displayed as spidergram, allows multiple responses to be overlaid.	1) Each team member completes online assessment individually–provides justification and confidence rating<br>2) Consensus process to agree on final rating.	Tool is entirely internet based and requires low level of effort from any external partner.	Internally-driven assessment.<br><br>Brings together multiple practice members and provides venue for recognizing and discussing discrepancies.
**JEE**	http://apps.who.int/iris/bitstream/handle/10665/204368/9789241510172_eng.pdf?sequence=1&isAllowed=y	Joint external evaluation	Monitoring and evaluation of progress towards International Health Regulations	PDF tool.<br><br>Approximately 50 indicators grouped into 4 domains. Each indicator assessed on scale of 0 (No Capacity) to 5 (Sustainable Capacity).	1) Self-evaluation. Countries request to complete evaluation, and work with relevant stakeholders to conduct self-assessment across the technical areas. Stakeholders asked to provide all information necessary, including supporting documentation where available.<br>2) External Evaluation. Country shares self-assessment with JEE team (multi-sectoral team of experts from member states, WHO, other international organizations). Five-day meeting where hosts present self-evaluation results (all relevant stakeholders present again). JEE team works with country officials to assign scores. May conduct site visits. Preliminary results presented to high-level stakeholders on final day of trip. Report produced within two weeks, shared with country for feedback, and then posted online.	Resources to organize meetings, sensitize stakeholders, and facilitate external partnership on assessments.	Standardized process across countries. External scoring characterized by multistakeholder approach and transparency and opennes of data sharing to allow for prioritization and resource allocation.
**PEPFAR Sustainability Indices and Dashboard 2.0**	https://www.pepfar.gov/countries/cop/c71524.htm	Joint external evaluation	Sustainability of national HIV/AIDS responses	PDF tool.<br><br>90 indicators grouped into 15 domains. Each domain can score up to 10 points. Scoring is automatic within PDF.	Variations in implemenation by country. Three options listed in official documentation include:<br>Option 1: Joint Participation (Preferred). The PEPFAR Team/COM, or other development partner such as UNAIDS, would invite relevant government counterparts, donors, and civil society to a full day meeting. The meeting is facilitated by a professional facilitator. Existing data sources required to answer the SID questions are gathered in advance and made available for the meeting. PEPFAR gives a presentation on the Sustainability Index and Dashboard. The group is divided into five working groups, one per domain. Each group answers all the questions under that domain, using existing data. If the data has not been made available, the participants can offer a response citing other existing documents and reliable data. The data source would need to be confirmed. Once the five groups have completed their domain, all the responses are put into one country-specific excel-based tool. The dashboard will be auto-generated. The dashboard would be shared with the wider group. Discussions on the findings and implications for investing in the weak elements would then be held in a large group. The larger group would discuss which elements are priorities for investment and which partner(s) – the government, a multilateral agency, other bilateral, PEPFAR, or private foundation – is best placed to provide technical and financial resources to strengthen that element. The Dashboard can then be used in high level diplomatic or technical dialogue to determine PEPFAR investments for COP 15.<br>Option 2: PEPFAR and development partners do a trial run. In this approach, the government and civil society are not initially engaged until after the Index is completed by a group comprised of development partners, including PEFPAR. Once the Index is completed, the government and civil society are engaged in a process of completing the Index with development partners. Any disagreements are checked against existing evidence. Then the process of reviewing and discussions ensues as per above. The benefit of this approach is that evidence and data can be collected in advance.<br>Option 3: Internal USG Approach. In contexts where there are significant sensitivities, the PEPFAR Team and Embassy Personnel may want to complete the Index and confer with other key stakeholders and civil society in a less formal setting. In this setting, it will be up to the PEPFAR leadership in country how to use the findings with government partners.	Resources to organize meetings, sensitize stakeholders, and facilitate external partnership on assessments.	Process allowed to vary by country depending on culture, technical capacity, time constraints, etc.<br><br>Multistakeholder engagement is ideal, if situation allows.
**GFATM Transition Readiness Assessment Tool**	https://eecaplatform.org/en/transition-readiness-assessment-tool-trat/	External	A country's readiness for, and risks of, transition from donor funding to sustainable domestic financing	Excel tool.<br><br>12 indicators grouped into four thematic areas. Each indicator assessed against three scaled benchmarks (Stage 1, 2, and 3).	1) Desk review<br>2) Key Informant Interviews (interview guides provided for government partners, CSO, technical partners, and donor agencies)<br>3) Assessors summarize/synthesize and make judgement about which benchmarks have or have not been achieved. Excel document to capture progress against benchmarks and automatically calculate readiness score		
**Patient-Centered Medical Home Assessment Tool**	http://www.safetynetmedicalhome.org/sites/default/files/PCMH-A.pdf	Self	Individual site's "medical homeness" and progress towards practice transformation.	PDF tool.<br><br>36 items grouped into eight "change concepts." Each item is assessed on a scale from 1-12, where 1-3 correspond to "Level D", 4-6 correspond to "Level C", 7-9 correspond to "Level B", and 10-12 correspond to "Level A."	Every site forms a multi-disciplinary assessment team. Ideally, each team member completes an assessment individually, and then teams meet to develop a consensus assessment. Asessments can be reviewed/validated by an external group.	Tool requires low level of effort from any external partner.	Multidisciplinary team is critical to assessment.
**12 Components Monitoring and Evaluation System Strengthening Tool (MEASURE and UNAIDS)**	www.unaids.org/en/media/unaids/contentassets/documents/document/2010/2_MERG_Strengthening_Tool_12_Components_ME_System.pdf	varies	National-level HIV Monitoring and Evaluation systems	Series of statements grouped into 12 components. Three possible response scales:<br>1. a 5-point scale (Yes-completely, Mostly, Partly, No-not at all, Not Applicable)<br>2. a 3-point scale (Yes, No, Not Applicable)<br>3. numerical responses	Method of implementation varied by context:<br>-Some countries used as a self-assessment for establishing a baseline<br>-Often donor organizations or technical partners would lead assessment process to prioritize within potential intervention areas<br><br>Typically, assessment would be implemented through a workshop bringing together all relevant government, development partner, and funder stakeholders. Participants would speak from their experience, backing up with evidence and/or documentation if available.	Resources to organize meetings, sensitize stakeholders, and facilitate external partnership on assessments.	Assessment can be a capacity-building intervention in and of itself. Valuable to have interactive, multidisciplinary workshops to get all stakeholders on the same page with shared understanding.
